# The Effect of Exenatide on Cardiovascular Risk Markers in Women With Polycystic Ovary Syndrome

**DOI:** 10.3389/fendo.2019.00189

**Published:** 2019-04-02

**Authors:** Alison J. Dawson, Thozhukat Sathyapalan, Rebecca Vince, Anne-Marie Coady, Ramzi A. Ajjan, Eric S. Kilpatrick, Stephen L. Atkin

**Affiliations:** ^1^Department of Diabetes and Endocrinology, University of Hull, Hull, United Kingdom; ^2^Department of Academic Endocrinology, Diabetes and Metabolism, Hull York Medical School, Heslington, United Kingdom; ^3^Department of Sports Science, University of Hull, Hull, United Kingdom; ^4^Department of Ultrasound, Hull and East Yorkshire Women's and Children's Hospital, Hull, United Kingdom; ^5^Division of Cardiovascular and Diabetes Research, Leeds Institute for Genetics, Health and Therapeutics, University of Leeds, Leeds, United Kingdom; ^6^Sidra Medical Research Centre, Doha, Qatar; ^7^Weill Cornell Medicine Qatar, Education City, Doha, Qatar; ^8^Royal College of Surgeons in Ireland (Bahrain), Al Muharraq, Bahrain

**Keywords:** polycystic ovary syndrome, exenatide, cardiovascular risk, endothelial function, inflammation, blood clot function, GLP-1 receptor agonists

## Abstract

**Background:** Polycystic ovary syndrome (PCOS) is associated with an adverse cardiovascular risk profile including a prothrombotic state. Exenatide has been shown to be effective at improving insulin sensitivity and weight loss in PCOS; therefore this study was undertaken to assess its effects on weight, endothelial function, inflammatory markers, and fibrin structure/function in overweight/obese women with PCOS.

**Methods:** Thirty overweight/obese anovulatory women with all 3 Rotterdam criteria received exenatide 5 mcg bd for 4 weeks then 10 mcg bd for 12 weeks. The primary outcome was change in weight; secondary outcomes were changes in endothelial function [Reactive Hyperemia-Peripheral Arterial Tonometry (RH-PAT)], serum endothelial markers (ICAM-1, VCAM-1, E-selectin, and P-selectin), change in inflammation (hsCRP), and alteration in clot structure and function [maximum absorbance (MA), and time from full clot formation to 50% lysis (LT)].

**Results:** Twenty patients completed the study. Exenatide reduced weight 111.8 ± 4.8 to 108.6 ± 4.6 kg *p* = 0.003. Serum endothelial markers changed with a reduction in ICAM-1 (247.2 ± 12.9 to 231.3 ± 11.5 ng/ml *p* = 0.02), p-selectin (101.1 ± 8.2 to 87.4 ± 6.6 ng/ml *p* = 0.01), and e-selectin (38.5 ± 3.3 to 33.6 ± 2.6 ng/ml *p* = 0.03), without an overt change in endothelial function. Inflammation improved (CRP; 8.5 ± 1.4 to 5.6 ± 0.8 mmol/L *p* = 0.001), there was a reduction in clot function (LT; 2,987 ± 494 to 1,926 ± 321 s *p* = 0.02) but not clot structure.

**Conclusion:** Exenatide caused a 3% reduction in weight, improved serum markers of endothelial function, inflammation, and clot function reflecting an improvement in cardiovascular risk indices in these women with PCOS. This suggests exenatide could be an effective treatment for obese women with PCOS.

**Clinical Trial Registration:** ISRCTN81902209.

## Introduction

Polycystic ovary syndrome (PCOS) is a common condition affecting 10% of women of reproductive age (1) and is characterized by chronic anovulation and hyperandrogenism.

Women with PCOS have an adverse cardiovascular risk profile including obesity, hypertension, dyslipidaemia, impaired glucose tolerance, and ischemic heart disease (2–4). Endothelial dysfunction is an early marker of atherosclerosis and has been shown to be present in women with PCOS in some (5–7) but not in all studies (8, 9). Treatments resulting in an improvement in endothelial function may slow the progression of atherosclerosis and therefore may reduce the risk of future cardiovascular events (10) and an improvement of endothelial function has been shown with weight loss and metformin treatment in women with PCOS (11). Serological markers of endothelial function include endothelin-1 that is reported to be elevated in PCOS (12), but decreased after 6 months metformin therapy (12). The endothelial function marker intercellular adhesion molecule-1 (ICAM-1) has also been reported to be elevated in PCOS (13).

Inflammatory markers have been noted to be higher in patients with PCOS; C-reactive protein (CRP) has consistently been shown to be greater in PCOS than controls (14, 15). PCOS has been confirmed to be a prothrombotic state compared to controls (16) with raised levels of plasminogen activator inhibitor 1 (PAI-1) activity and fibrinogen being reported.

Abnormal clot structure and function contributes to the prothrombotic state; clots composed of a compact structure with thin fibers and small pores are associated with early and more severe cardiovascular disease (17). Clot structure is denser and less porous in individuals with type 2 diabetes (18) compared to controls, and has been shown to be less permeable in healthy relatives of people with premature coronary artery disease (19). Metformin has been shown to be beneficial in altering clot structure and function in patients with type 2 diabetes (20) that may reflect reduced insulin resistance.

Exenatide, an incretin mimetic, is a glucagon like polypeptide (GLP-1) analog used in the treatment of type 2 diabetes (21). It has several methods of action including enhancing glucose-dependent insulin synthesis from pancreatic beta cells, decreasing glucagon production, and slowing gastric emptying time. GLP-1 analogs have shown to reduce weight (22–27), reduce hyperandrogenism (22, 27) and improve menstrual cyclicity (22, 25) in women with PCOS. In one study in patients with PCOS, exenatide with or without metformin was given for 6 months and showed an improvement in menstrual frequency and hyperandrogenism (22). There was a reduction in the anti-inflammatory marker adiponectin, but no change in CRP, interleukin-6 (IL-6), or tumor-necrosis factor-α (TNFα). In a study examining obese patients with type 2 diabetes exenatide, given for 12 weeks was shown to exert a potent anti-inflammatory effect at the cellular and molecular level (28).

We therefore hypothesized that exenatide would exert a direct effect on endothelial function, inflammatory markers and blood clot structure and function in overweight/obese women with PCOS.

## Patients and Methods

An open labeled study was undertaken using exenatide 5 micrograms twice daily for 1 month followed by exenatide 10 μg twice daily for 3 months. The diagnosis of PCOS was based on all three diagnostic criteria of the Rotterdam consensus being present for each patient to reduce the heterogeneity of the diagnosis, namely clinical, and biochemical evidence of hyperandrogenemia [Ferriman-Gallwey score >8; free androgen index (FAI) >4], oligomenorrhoea or amenorrhea, and polycystic ovaries on transvaginal ultrasound. Non-classical 21-hydroxylase deficiency, hyperprolactinaemia, Cushing's disease, and androgen-secreting tumors were excluded by appropriate tests. Subjects had no concurrent illness, were not on any prescription or over-the-counter medication that was likely to affect insulin sensitivity, lipids, or ovarian function including hormonal contraceptives for the preceding 6 months. Subjects were not planning to conceive and were using barrier contraception. Subjects were advised not to change their lifestyle, including physical activity, or dietary habits, during the study period.

Thirty patients (mean age 27 ± 4 years) who fulfilled both inclusion and exclusion criteria were given exenatide. Compliance with treatment was calculated by determining the amount of exenatide in the pens returned at the next clinic visit (2 and 4 months).

All subjects gave written informed consent in accordance with the Declaration of Helsinki. The protocol was approved by the Leeds East Local Research Ethics committee.

Clinical and fasting biochemical assessments were performed at baseline and at the end of the 4-month period. The primary endpoint of the study was a change in weight, and the secondary endpoints were changes in endothelial function, inflammation, and clot structure.

Fasting venous blood was collected into serum gel and fluoride oxalate tubes. Samples were separated by centrifugation at 2,000 × *g* for 15 min at 4°C, and the aliquots stored at −20°C. Serum testosterone was measured by liquid chromatography/mass spectroscopy on an Acquity UPLC system coupled to a Quattro Premier XE mass spectrometer (Waters, Manchester, UK). Sex hormone binding globulin (SHBG) was measured by an immunometric assay with fluorescence detection on the DPC Immulite 2000 analyzer using the manufacturer's recommended protocol (upper limit of the reference range 2.0 nmol/l). The FAI was calculated as the total testosterone × 100/SHBG. Total cholesterol, triglycerides, and high-density lipoprotein cholesterol (HDL-C) levels were measured enzymatically using a Synchron LX20 analyzer (Beckman-Coulter, High Wycombe, UK). Low-density lipoprotein cholesterol (LDL-C) was calculated using the Friedewald equation. Serum insulin was assayed using a competitive chemiluminescent immunoassay performed on the DPC Immulite 2000 analyzer (Euro/DPC, Llanberis, UK). The analytical sensitivity of the insulin assay was 2 μU/ml, the coefficient of variation was 6%, and there was no stated cross-reactivity with proinsulin. Plasma glucose was measured using a Synchron LX 20 analyzer (Beckman-Coulter), using the manufacturer's recommended protocol. The coefficient of variation for the assay was 1.2% at a mean glucose value of 5.3 mmol/L during the study period. The insulin resistance was calculated using the homeostasis model assessment for insulin resistance (HOMA) method [HOMA-IR = (insulin x glucose)/22.5]. Alanine aminotransferase was measured on the Unicel® DxC 80 analyser (Beckamn-Coulter, High Wycome, UK).

The serum concentrations of serum intercellular adhesion molecule 1 (ICAM-1), serum vascular cell adhesion molecule 1 (sVCAM-1), and sE-selectin were measured by commercially available quantitative ELISA (R&D Systems, USA) according to the manufacturer's instructions.

Reactive hyperemia peripheral arterial tonometry (RH-PAT) is a non-invasive technique to assess peripheral microvascular endothelial function by measuring changes in digital pulse volume during reactive hyperemia. The RH-PAT data were analyzed by a computer in an operator-independent manner as previously described (29). The RH-PAT index was calculated as the ratio of the average amplitude of the PAT signal over a 1-min time interval starting 1 min after cuff deflation divided by the average amplitude of the PAT signal of a 3.5-min time period before cuff inflation (baseline). This was carried out after 12 h fast at 9 a.m. in a stable environment (20°C air conditioned room having allowed for an equilibrium period of 15 min with dimmed lights according to manufacturer's instructions) (29).

Clot structure and fibrinolysis measurements were conducted using turbidity and lysis measurements as previously described (30). In brief, for turbidity measurements, plasma samples (25 μl) were added to a 96 well-Greiner plate (in duplicate) with 50 mmol^−1^ Tris, 100 mmol^−1^ NaCl pH 7.4 (75 μl). Activation mix (50 μl) containing 0.045 U ml^−1^ human thrombin (Calbiochem), and 22.5 mmol^−1^ calcium in assay buffer was added to each column with a multi-channel pipette at 10-s intervals. Plates were shaken and read at 340 nm every 12 s for 60 min in an ELx-808 IU ultramicroplate reader (BIO-TEK Instruments INC, USA).

For lysis measurements, the same procedure was applied, but with the addition of 333 ng ml^−1^ tissue plasminogen activator (tPA) (75 μl) (Technoclone, Vienna, Austria). A number of parameters were subsequently analyzed including final turbidity (FT), time from initiation of clot formation to 50% lysis (LT1) and time from start of clot formation to 50% lysis (LT2).

### Statistical Analysis

The sample size was based on the study of Elkind-Hirsch et al treating women with PCOS on the effect of exenatide on weight loss in women with PCOS (22). Using nQuery advisor sample size software, for 80% power and a significance level of 5%, a sample size of 20 was calculated. Adjusting for a possible 30% dropout rate due to side effects reported (22) a total of 30 patients were recruited.

A comparison between the groups at baseline and follow up was carried out using the paired *t*-test for biochemical data and clinical observations. The Wilcoxon signed rank test was applied to biochemical data that violated the assumptions of normality when tested using the Kolmogorov–Smirnov test, and associations used Pearson's correlation or Spearman's correlation as appropriate. Statistical analysis was performed using SPSS for Windows NT, version 18.0 (SPSS Inc., Chicago, IL). Data are reported as mean ± SEM.

## Results

Twenty patients aged 26 ± 4 years completed the study. One patient became pregnant during the study, and six patients withdrew due to side effects [nausea, vomiting, and dizziness (two patients on the 5 μg twice daily dose and four patients on the 10 μg twice daily dose)]. Two patients were withdrawn due to poor compliance during the first month and one patient was lost to follow up. The pregnancy was followed to term with no maternal or fetal complications.

The mean weight of the women at baseline was 110.0 ± 3.4 kg with BMI 40.4 ± 1.3 kg/m^2^ [3 women were overweight (BMI 26–29) whilst 27 were obese (BMI 30 or greater)] Systolic blood pressure was 119.7 ± 2.2 mmHg and diastolic was 71.5 ± 1.6 mmHg.

There was a significant reduction in weight from 111.8 ± 4.8 to 108.6 ± 4.6 kg (*p* = 0.003) and BMI 41.0 ± 1.9 to 39.8 ± 1.8 kg/m^2^ (*p* = 0.005). There was no effect on waist circumference but there was a reduction in hip circumference. Blood pressure was unaffected by the exenatide treatment (Table 1).

**Table 1:** Physical characteristics for the 20 subjects with PCOS before and after exenatide therapy (mean ± SD).

	**Baseline *N* = 20**	**After therapy *N* = 20**	***p*-value**
Weight (kg)	111.8 ± 4.8	108.6 ± 4.6	0.003
BMI (kg/m^2^)	41.0 ± 1.9	39.8 ± 1.8	0.005
Waist (cm)	120.2 ± 3.6	118.1 ± 4.1	0.31
Hip (cm)	129.3 ± 3.1	126.3 ± 3.2	0.001
Systolic blood pressure (mmHg)	118.1 ± 2.9	119.2 ± 3.2	0.60
Diastolic blood pressure (mmHg)	73.0 ± 1.8	73.3 ± 2.3	0.86
RH-PAT	1.67 ± 0.1	1.49 ± 0.1	0.06.

*BMI, body mass index; RH-PAT, reactive hyperemia-peripheral arterial tonometry*.

There was no change in FAI or SHBG at the completion of the study.

There was no effect on total cholesterol, HDL or LDL-cholesterol but there was an improvement in triglycerides 1.3 ± 0.1 to 1.2 ± 0.1 mmol/L *p* = 0.04. There was no difference in glucose, insulin and HOMA-IR comparing before and after exenatide treatment (Table 2).

**Table 2:** Laboratory data for the 20 subjects with PCOS before and after exenatide therapy.

	**Baseline *n* = 20**	**After therapy *n* = 20**	***p*-value**
ALT (IU/L)	32.5 ± 2.9	32.2 ± 3.3	0.84
CRP (mmol/L)	8.5 ± 1.4	5.6 ± 0.9	0.001
TC (mmol/L)	4.4 ± 0.2	4.5 ± 0.2	0.30
HDL-C (mmol/L)	1.08 ± 0.10	0.98 ± 0.07	0.22
LDL-C (mmol/L)	3.03 ± 0.14	2.98 ± 0.17	0.67
Triglycerides (mmol/L)	1.34 ± 0.14	1.17 ± 0.11	0.037
Fasting glucose (mmol/L)	5.0 ± 0.1	4.8 ± 0.1	0.24
Insulin (uIU/mL)	18.4 ± 2.7	18.8 ± 2.8	0.90
HOMA-IR	4.13 ± 0.64	4.07 ± 0.67	0.94
FAI (%)	15.8 ± 1.9	14.2 ± 1.9	0.36
SHBG (nmol/L)	23.4 ± 1.7	25.1 ± 1.9	0.36
VCAM-1 (ng/ml)	2284.3 ± 75.4	2329.8 ± 77.0	0.42
ICAM-1 (ng/ml)	247.2 ± 12.9	231.3 ± 11.5	0.02
E-selectin (ng/ml)	38.5 ± 3.3	33.6 ± 2.6	0.03
P-selectin (ng/ml)	101.1 ± 8.2	87.4 ± 6.6	0.01
LT1 (s)	3506 ± 489	2469 ± 307	0.02
LT2 (s)	2987 ± 494	1926 ± 321	0.02

*ALT, alanine aminotransferase; CRP, high sensitivity-C-reactive protein; TC, total cholesterol; LDL, low density lipoproteins; HDL, high density lipoproteins; HOMA-IR, homeostatic model assessment of Insulin resistance; FAI, free androgen index; SHBG, sex hormone binding globulin; VCAM, vascular cell adhesion molecule 1; ICAM, intercellular adhesion molecule 1; LT, lysis time. Data are presented as mean ± SD*.

Endothelial function determined by RH-PAT was unchanged [RH-PAT (1.67 ± 0.1 to 1.49 ± 0.1 *p* = 0.06)]; however, the surrogate measures of endothelial function improved with a reduction in ICAM-1 (247.2 ± 12.9 to 231.3 ± 11.5 ng/ml *p* = 0.02), p-selectin (101.1 ± 8.2 to 87.4 ± 6.6 ng/ml *p* = 0.01), and e-selectin (38.5 ± 3.3 to 33.6 ± 2.6 ng/ml *p* = 0.03). There was no change in VCAM-1.

There was an improvement in CRP from 8.5 ± 1.4 to 5.6 ± 0.8 mmol/L *p* = 0.001.

For clot formation there was a reduction in time from full clot formation to 50% lysis (LT) from 2,987 ± 494 to 1,926 ± 321 s *p* = 0.02, but exenatide did not have an effect on the maximum absorbance (MA).

There was a correlation between a change in ICAM and triglycerides (*r* = −0.59 *p* = 0.02), change in p-selectin and hip circumference (*r* = 0.61 *p* = 0.009) and change in CRP and insulin/HOMA (*r* = −0.57 *p* = 0.02 *r* = −0.49 *p* = 0.047). There was also a correlation between change in LT and systolic blood pressure (*r* = −0.63, *p* = 0.03).

The study time was too short to assess an effect on menstruation.

## Discussion

This study confirmed the modest weight loss that could be achieved with exenatide in overweight women with PCOS with improved serum markers of endothelial function, inflammation, and improved clot function, all of which reflected an improvement in cardiovascular risk indices in these women with PCOS.

In accord with a previous study on GLP-1 analogs (31), exenatide was found to reduce weight (an average of 3.4 kg over 16 weeks). This is consistent with values for weight loss in patients with diabetes being treated with exenatide [between 2 and 6 kg (32) for a duration of exenatide between 90 and 365 days (33)]. In PCOS patients, the mean weight loss was 3.2 kg after a total of 16 weeks of exenatide treatment (5 mcg bd for 4 weeks then 10 mcg bd for 12 weeks). The patients in our study lost more weight than previously quoted that could be explained by the patients in our study having a higher BMI at baseline (average BMI 41.0 kg/m^2^) compared to other studies. The study was too short to determine changes in menstrual frequency though increased menstrual frequency with exenatide has been shown by others (34).

The endothelium is crucial in regulating vascular function by modulating tone, growth, homeostasis, and inflammation (35). The endothelium has several roles including endothelium dependent vasodilatation, regulation of cellular adhesion molecules, and releasing key homeostatic regulatory molecules (36). The cellular adhesion markers have previously been shown to be raised in PCOS (12, 13) and therefore the reductions of ICAM-1, e-selectin and p-selectin that were seen in our study may suggest the mechanism of how exenatide improves the adverse cardiovascular risk profile in PCOS. However, the inconsistency between the results of endothelial function and serum endothelial markers seen in our study is in accord with that reported by others in PCOS (5–9). There was a positive change in the endothelial markers, but whilst there was a trend, there was the inconsistency that endothelial function did not significantly improve when measured by the Endopat2000. This may highlight that the serological cellular adhesion molecules may be modifiable with 16 weeks of exenatide treatment whereas RH-PAT may require more time to show a significant change, and clarification of these functional findings with longer studies involving serial measurement of the serum endothelial markers are required. The correlation of decreased ICAM-1 with triglycerides and p-selectin with hip circumference is in accord with their relationship with metabolic syndrome and their reduction that reflected the weight loss (37, 38).

Another explanation for the inconsistency in endothelial function may be the different methods used to calculate endothelial function, although an advantage of the Endopat2000 is that it is operator independent (29). Exenatide has been shown to have a short term benefit on endothelial function in patients with type 2 diabetes (39) (using RH-PAT) when measured after one injection of exenatide or placebo but long term effects on endothelial function have not been reported.

Endothelial function is an early marker of cardiovascular disease whereas blood clot structure and function is a late marker. A novel finding in this study was that exenatide resulted in a reduction of time from full clot formation to 50% lysis (LT) which may be relevant as an increase in LT has been shown to be associated with cardiovascular risk (30); these findings need to be confirmed as this may add a new facet to the cardiovascular risk that these women have. The correlation of LT to systolic blood pressure likely reflects the complex relationship of the fibrinolytic potential and arterial structure and function (40). An adverse cardiovascular risk profile has been demonstrated in PCOS(2) although evidence for increased cardiovascular mortality is not well-established (41). More angiographic coronary artery disease and worsening cardiovascular event free survival has been shown in postmenopausal women with a history of PCOS compared to those without a PCOS history (42).

An improvement in inflammation was seen in our study with a significant reduction in both CRP and serum triglycerides. This is in accord with reports of exenatide use in patients with type 2 diabetes (43, 44), but the reduction in CRP was not seen in a previous study with PCOS patients (22); however, baseline CRP was measured using different methods between the two studies and cannot be compared, though our patients had a higher level of triglycerides (4.4 vs. 1.16 nmol/L) that may explain the significant reduction. Insulin resistance correlated with the reduction in CRP as well-described by others (45).

In contrast to a previous study with exenatide our study showed there was no reduction in total testosterone or HOMA (22) that may be explained by the difference in duration of the study (our study was shorter at 4 months compared to 6 months) and the different patient population studied.

The weakness of this study is the high drop-out rate which largely was due to side effects of the medication (namely nausea and vomiting), however the withdrawal rate was similar to that reported in a previous study (22) of 30%. An additional limitation was the lack of a group of controls such as obese women not affected by PCOS to determine whether those changes reported were independent of the PCOS phenotype.

In summary, this study has shown that exenatide is effective in improving the parameters of adverse cardiovascular risk in obese women with PCOS. There was no change of endothelial function by direct measurement that may indicate that surrogate markers do not reflect endothelial change in PCOS.

## Data Availability

The datasets generated for this study are available on request to the corresponding author.

## Author Contributions

AD performed the study. RV performed and interpreted the serum endothelial markers. A-MC performed the radiology. RA supervised the clotting studies. TS, EK, and SA supervised the study. All authors contributed to writing the manuscript.

### Conflict of Interest Statement

The authors declare that the research was conducted in the absence of any commercial or financial relationships that could be construed as a potential conflict of interest.

## References

[1] Dunaif A. Insulin resistance and the polycystic ovary syndrome: mechanism and implications for pathogenesis. Endocr Rev. (1997) 18:774–800. 10.1210/er.18.6.774 9408743

[2] Lo JC Feigenbaum SL Yang J Pressman AR Selby JV Go AS. Epidemiology and adverse cardiovascular risk profile of diagnosed polycystic ovary syndrome. J Clin Endocrinol Metab. (2006) 91:1357–63. 10.1210/jc.2005-2430 16434451

[3] Marchesini G Bugianesi E Forlani G Cerrelli F Lenzi M Manini R. Nonalcoholic fatty liver, steatohepatitis, and the metabolic syndrome. Hepatology. (2003) 37:917–23. 10.1053/jhep.2003.50161 12668987

[4] Hart R Doherty DA. The potential implications of a PCOS diagnosis on a woman's long-term health using data linkage. J Clin Endocrinol Metab. (2015) 100:911–9. 10.1210/jc.2014-3886 25532045

[5] Sorensen MB Franks S Robertson C Pennell DJ Collins P. Severe endothelial dysfunction in young women with polycystic ovary syndrome is only partially explained by known cardiovascular risk factors. Clin Endocrinol. (2006) 65:655–9. 10.1111/j.1365-2265.2006.02645.x 17054469

[6] Tarkun I Arslan BC Canturk Z Turemen E Sahin T Duman C. Endothelial dysfunction in young women with polycystic ovary syndrome: relationship with insulin resistance and low-grade chronic inflammation. J Clin Endocrinol Metab. (2004) 89:5592–6. 10.1210/jc.2004-0751 15531516

[7] Kravariti M Naka KK Kalantaridou SN Kazakos N Katsouras CS Makrigiannakis A. Predictors of endothelial dysfunction in young women with polycystic ovary syndrome. J Clin Endocrinol Metab. (2005) 90:5088–95. 10.1210/jc.2005-0151 15985492

[8] Mather KJ Verma S Corenblum B Anderson TJ. Normal endothelial function despite insulin resistance in healthy women with the polycystic ovary syndrome. J Clin Endocrinol Metab. (2000) 85:1851–6. 10.1210/jc.85.5.1851 10843164

[9] Brinkworth GD Noakes M Moran LJ Norman R Clifton PM. Flow-mediated dilatation in overweight and obese women with polycystic ovary syndrome. BJOG. (2006) 113:1308–14. 10.1111/j.1471-0528.2006.01090.x 17059392

[10] Celermajer DS. Endothelial dysfunction: does it matter? Is it reversible? J Am Coll Cardiol. (1997) 30:325–33. 10.1016/S0735-1097(97)00189-7 9247501

[11] Agarwal N Rice SP Bolusani H Luzio SD Dunseath G Ludgate M. Metformin reduces arterial stiffness and improves endothelial function in young women with polycystic ovary syndrome: a randomized, placebo-controlled, crossover trial. J Clin Endocrinol Metab. (2010) 95:722–30. 10.1210/jc.2009-1985 19996308

[12] Diamanti-Kandarakis E Spina G Kouli C Migdalis I. Increased endothelin-1 levels in women with polycystic ovary syndrome and the beneficial effect of metformin therapy. J Clin Endocrinol Metab. (2001) 86:4666–73. 10.1210/jcem.86.10.7904 11600523

[13] Nasiek M Kos-Kudla B Ostrowska Z Marek B Kajdaniuk D Sieminska L. Plasma concentration of soluble intercellular adhesion molecule-1 in women with polycystic ovary syndrome. Gynecol Endocrinol. (2004) 19:208–15. 10.1080/09513590400014313 15724804

[14] Boulman N Levy Y Leiba R Shachar S Linn R Zinder O. Increased C-reactive protein levels in the polycystic ovary syndrome: a marker of cardiovascular disease. J Clin Endocrinol Metab. (2004) 89:2160–5. 10.1210/jc.2003-031096 15126536

[15] Verit FF. High sensitive serum C-reactive protein and its relationship with other cardiovascular risk factors in normoinsulinemic polycystic ovary patients without metabolic syndrome. Arch Gynecol Obstet. (2010) 281:1009–14. 10.1007/s00404-009-1226-6 19771438

[16] Manneras-Holm L Baghaei F Holm G Janson PO Ohlsson C Lonn M. Coagulation and fibrinolytic disturbances in women with polycystic ovary syndrome. J Clin Endocrinol Metab. (2011) 96:1068–76. 10.1210/jc.2010-2279 21252248

[17] Fatah K Silveira A Tornvall P Karpe F Blomback M Hamsten A. Proneness to formation of tight and rigid fibrin gel structures in men with myocardial infarction at a young age. Thromb Haemost. (1996) 76:535–40. 10.1055/s-0038-1650618 8902992

[18] Dunn EJ Ariens RA Grant PJ. The influence of type 2 diabetes on fibrin structure and function. Diabetologia. (2005) 48:1198–206. 10.1007/s00125-005-1742-2 15864538

[19] Mills JD Ariens RA Mansfield MW Grant PJ. Altered fibrin clot structure in the healthy relatives of patients with premature coronary artery disease. Circulation. (2002) 106:1938–42. 10.1161/01.CIR.0000033221.73082.06 12370216

[20] Grant PJ. Beneficial effects of metformin on haemostasis and vascular function in man. Diabetes Metab. (2003) 29:6S44–52. 14502100 10.1016/s1262-3636(03)72787-6

[21] Egan JM Clocquet AR Elahi D. The insulinotropic effect of acute exendin-4 administered to humans: comparison of nondiabetic state to type 2 diabetes. J Clin Endocrinol Metab. (2002) 87:1282–90. 10.1210/jcem.87.3.8337 11889200

[22] Elkind-Hirsch K Marrioneaux O Bhushan M Vernor D Bhushan R. Comparison of single and combined treatment with exenatide and metformin on menstrual cyclicity in overweight women with polycystic ovary syndrome. J Clin Endocrinol Metab. (2008) 93:2670–8. 10.1210/jc.2008-0115 18460557

[23] Rasmussen CB Lindenberg S. The effect of liraglutide on weight loss in women with polycystic ovary syndrome: an observational study. Front Endocrinol. (2014) 5:140. 10.3389/fendo.2014.00140 25221543 PMC4145240

[24] Kahal H Abouda G Rigby AS Coady AM Kilpatrick ES Atkin SL. Glucagon-like peptide-1 analogue, liraglutide, improves liver fibrosis markers in obese women with polycystic ovary syndrome and nonalcoholic fatty liver disease. Clin Endocrinol. (2014) 81:523–8. 10.1111/cen.12369 24256515

[25] Jensterle M Salamun V Kocjan T Vrtacnik Bokal E Janez A. Short term monotherapy with GLP-1 receptor agonist liraglutide or PDE 4 inhibitor roflumilast is superior to metformin in weight loss in obese PCOS women: a pilot randomized study. J Ovarian Res. (2015) 8:32. 10.1186/s13048-015-0161-3 26032655 PMC4506413

[26] Jensterle M Kocjan T Kravos NA Pfeifer M Janez A. Short-term intervention with liraglutide improved eating behavior in obese women with polycystic ovary syndrome. Endocr Res. (2015) 40:133–8. 10.3109/07435800.2014.966385 25330463

[27] Jensterle M Goricar K Janez A. Metformin as an initial adjunct to low-dose liraglutide enhances the weight-decreasing potential of liraglutide in obese polycystic ovary syndrome: randomized control study. Exp Ther Med. (2016) 11:1194–200. 10.3892/etm.2016.3081 27073422 PMC4812364

[28] Chaudhuri A Ghanim H Vora M Sia CL Korzeniewski K Dhindsa S. Exenatide exerts a potent antiinflammatory effect. J Clin Endocrinol Metab. (2012) 97:198–207. 10.1210/jc.2011-1508 22013105 PMC3251936

[29] Bonetti PO Pumper GM Higano ST Holmes DR Jr Kuvin JT Lerman A. Noninvasive identification of patients with early coronary atherosclerosis by assessment of digital reactive hyperemia. J Am Coll Cardiol. (2004) 44:2137–41. 10.1016/j.jacc.2004.08.062 15582310

[30] Carter AM Cymbalista CM Spector TD Grant PJ. Heritability of clot formation, morphology, and lysis: the EuroCLOT study. Arterioscler Thromb Vasc Biol. (2007) 27:2783–9. 10.1161/ATVBAHA.107.153221 17932316

[31] Lamos EM Malek R Davis SN. GLP-1 receptor agonists in the treatment of polycystic ovary syndrome. Exp Rev Clin Pharmacol. (2017) 10:401–8. 10.1080/17512433.2017.1292125 28276778

[32] Dushay J Gao C Gopalakrishnan GS Crawley M Mitten EK Wilker E. Short-term exenatide treatment leads to significant weight loss in a subset of obese women without diabetes. Diabetes Care. (2012) 35:4–11. 10.2337/dc11-0931 22040840 PMC3241299

[33] Horton ES Silberman C Davis KL Berria R. Weight loss, glycemic control, and changes in cardiovascular biomarkers in patients with type 2 diabetes receiving incretin therapies or insulin in a large cohort database. Diabetes Care. (2010) 33:1759–65. 10.2337/dc09-2062 20460445 PMC2909058

[34] Liu X Zhang Y Zheng SY Lin R Xie YJ Chen H. Efficacy of exenatide on weight loss, metabolic parameters and pregnancy in overweight/obese polycystic ovary syndrome. Clin Endocrinol. (2017) 87:767–74. 10.1111/cen.13454 28834553

[35] Vita JA Keaney JF Jr. Endothelial function: a barometer for cardiovascular risk? Circulation. (2002) 106:640–2. 12163419 10.1161/01.cir.0000028581.07992.56

[36] Celermajer DS. Reliable endothelial function testing: at our fingertips? Circulation. (2008) 117:2428–30. 10.1161/CIRCULATIONAHA.108.775155 18474821

[37] Patel MS Miranda-Nieves D Chen J Haller CA Chaikof EL. Targeting P-selectin glycoprotein ligand-1/P-selectin interactions as a novel therapy for metabolic syndrome. Transl Res. (2017) 183:1–13. 10.1016/j.trsl.2016.11.007 28034759 PMC5393932

[38] Tabur S Oztuzcu S Oguz E Demiryurek S Dagli H Alasehirli B. Evidence for elevated (LIMK2 and CFL1) and suppressed (ICAM1, EZR, MAP2K2, and NOS3) gene expressions in metabolic syndrome. Endocrine. (2016) 53:465–70. 10.1007/s12020-016-0910-0 26956845

[39] Koska J Schwartz EA Mullin MP Schwenke DC Reaven PD. Improvement of postprandial endothelial function after a single dose of exenatide in individuals with impaired glucose tolerance and recent-onset type 2 diabetes. Diabetes Care. (2010) 33:1028–30. 10.2337/dc09-1961 20200309 PMC2858168

[40] Pieters M Boshuizen H de Lange Z Schutte AE Schutte R Greeff M. Relationship of coagulation and fibrinolytic variables with arterial structure and function in Africans. Thromb Res. (2014) 134:78–83. 10.1016/j.thromres.2014.04.021 24824291

[41] Wild RA. Polycystic ovary syndrome: a risk for coronary artery disease? Am J Obstet Gynecol. (2002) 186:35–43. 10.1067/mob.2002.119180 11810081

[42] Shaw LJ Bairey Merz CN Azziz R Stanczyk FZ Sopko G Braunstein GD. Postmenopausal women with a history of irregular menses and elevated androgen measurements at high risk for worsening cardiovascular event-free survival: results from the National Institutes of Health–National Heart, Lung, and Blood Institute sponsored Women's Ischemia Syndrome Evaluation. J Clin Endocrinol Metab. (2008) 93:1276–84. 10.1210/jc.2007-0425 18182456 PMC2291491

[43] Varanasi A Chaudhuri A Dhindsa S Arora A Lohano T Vora MR. Durability of effects of exenatide treatment on glycemic control, body weight, systolic blood pressure, C-reactive protein, and triglyceride concentrations. Endocr Pract. (2011) 17:192–200. 10.4158/EP10199.OR 20841306

[44] Gentilella R Bianchi C Rossi A Rotella CM. Exenatide: a review from pharmacology to clinical practice. Diab Obes Metab. (2009) 11:544–56. 10.1111/j.1463-1326.2008.01018.x 19383034

[45] Lorenzo C Festa A Hanley AJ Rewers MJ Escalante A Haffner SM. Novel protein glycan-derived markers of systemic inflammation and c-reactive protein in relation to glycemia, insulin resistance, and insulin secretion. Diabetes Care. (2017) 40:375–82. 10.2337/dc16-1569 28031420 PMC5319478

